# Differences in the dynamic balance function of healthy elementary school students and university students observed with and without the use of a sensor-integrated gamification application

**DOI:** 10.3389/fresc.2025.1680457

**Published:** 2025-11-12

**Authors:** Yasuaki Kusumoto, Satoko Ohmatsu, Eri Takahashi, Kanako Nakamura

**Affiliations:** 1 Department of Physical Therapy, Fukushima Medical University School of Health Sciences, Fukushima, Japan; 2 Digireha Inc., Tokyo, Japan

**Keywords:** dynamic balance function, healthy elementary school students, university students, sensor-integrated gamification application, pediatric rehabilitation

## Abstract

**Objective:**

This study examined differences in the dynamic balance function of healthy elementary school students and university students observed with and without the use of a sensor-integrated gamification application developed for pediatric rehabilitation.

**Methods:**

This cross-sectional study was conducted between January and June 2025. and included a total of 79 participants (43 healthy elementary school students from Fukuoka, Japan; 36 healthy university students from Fukushima, Japan). Measurements were performed using a normal Y-Balance test and a Y-Balance test with a sensor game to encourage slow movements. The sensor sensitivity was set to 0.768 g and 7.53 m/s^2^ during game play.

**Results:**

During the Y-Balance test, all items exhibited no significant interaction effects; however, several outcome measures exhibited main effects. The results of multiple comparison tests indicated different responses between groups attributable to sensor game use. No significant differences in the Y-Balance test items with or without the use of the sensor game for the dominant and non-dominant legs were observed in the university student group. Conversely, in the elementary school student group, the posterolateral scores of the Y-Balance test with the sensor game were significantly lower than those of the normal Y-Balance test.

**Conclusion:**

The sensor-integrated gamification application effectively increased task difficulty for healthy elementary school students by encouraging slower movements, leading to decreased dynamic balance function. This effect was not observed among university students. These findings suggest that sensor games may be valuable because they can appropriately adjust the difficulty level of balance exercises among elementary school students in rehabilitation settings.

## Introduction

Adjusting the difficulty of a task to the skill level of the learner to facilitate motor learning is important during rehabilitation (1). Various factors are relevant to adjusting the difficulty level of a task, including the speed, acceleration, and load intensity of the exercise (1, 2). In particular, when performing tasks that require muscle output and coordinated control of multiple joints, such as balance, if the difficulty level of the task is easy, then the desired effect cannot be achieved even if the task is performed more often (3, 4). The difficulty, task type, load setting, and number of repetitions must be appropriate to improve the dynamic balance function (5).

Among dynamic balance tasks, those that require slow and controlled movements are especially difficult because they require precise speed control and coordinated multijoint action (6). Moreover, the dynamic balance function is believed to mature at approximately 10–12 years of age (7, 8), thus underscoring the importance of age-appropriate task design.

Age-related changes in static and dynamic postural control of healthy children and adolescents suggest the need to tailor balance exercises based on their developmental stage (9, 10). Gamification may be effective for pediatric rehabilitation if it provides visual feedback to promote movements with consistent speed during balance training. However, few studies have investigated the difficulty levels of dynamic balance tasks using sensor technologies that can detect and quantify limb movements.

In pediatric populations, maintaining motivation to participate in rehabilitation that involves performing repetitive or monotonous training is difficult. Tasks that lack variety and complexity often fail to motivate learners. Therefore, gamification is gaining attention as an approach to promote participation in rehabilitation (11, 12). Gamification, which includes elements such as real-time feedback, scoring, and progressive adjustment of difficulty, enhances patient enjoyment, autonomy, and adherence in therapeutic settings (13, 14). Furthermore, gamification can facilitate repetitive practice in rehabilitation (15).

However, when these systems are intentionally designed to promote slower and more controlled movements by adjusting sensor sensitivity or timing settings, they can be used to purposefully increase task difficulty, thereby enhancing training effectiveness. As a result, a temporary decline in performance, particularly for children whose motor control systems are still developing, can occur. In contrast, university students with more mature motor function may be less affected.

Therefore, this study examined differences in the dynamic balance function of healthy elementary school students and university students observed with and without the use of a sensor-integrated gamification application developed for pediatric rehabilitation. We hypothesized that increasing task difficulty through higher sensor sensitivity would lead to a temporary decrease in dynamic balance performance among elementary school students but not among university students.

## Methods

**Figure 1:**
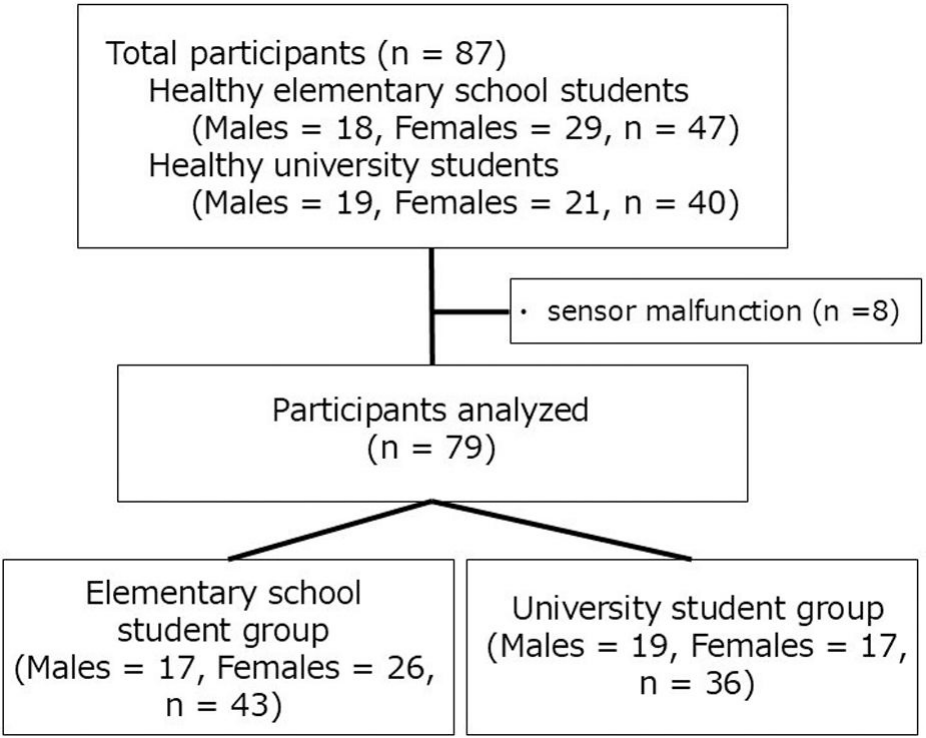
Participant flowchart.

This cross-sectional study was conducted between January 2025 and June 2025. A total of 87 participants were recruited; 47 participants were elementary school students (from Fukuoka City, Japan) and 40 participants were university students (from Fukushima City, Japan). Elementary school students were recruited from local after-school clubs, and university students were recruited from Fukushima Medical University. Eight participants (four each group) were excluded because sensor malfunctions that occurred during measurement prevented completion of the Y-Balance test. The final analysis included 79 participants (43 elementary school students and 36 university students) (Figure 1). A power calculation was conducted using G power to determine the required sample size, and the effect size was calculated using a moderate effect size based on Cohen's criterion (16). A repeated measures two-way analysis of covariance (ANCOVA) based on the criterion of the two-way analysis of variance (ANOVA) with the effect size set to 0.25, alpha level set to 0.05, and power set to 0.80 indicated that a total sample size of 36 was required.

This study was approved by the Ethical Review Committee of Fukushima Medical University (approval number: 2022-006). Written informed consent was obtained from all university students and from the guardians of the elementary school students.

### Y-Balance test

To measure dynamic postural control, we used the Y-Balance Test Kit^TM^ according to previously described methods (17, 18). The Y-Balance Test Kit^TM^ consists of three plastic pipes attached to a stance stand in the anterior reach, medial posterior reach, and lateral posterior reach directions. The posterior medial and posterior lateral pipes were located 135 degrees from the anterior pipe. The participants stood at the center of the footplate with the most distal point of the big toe on the starting line.

The participants were first measured while standing only on the dominant leg; then, they were measured while standing only on the non-dominant leg. Initially, the participants were instructed to push a target (reach indicator) along the pipe with the opposite leg (non-dominant leg) in three directions (anterior, posterior medial, and posterior lateral) to the maximum possible extent while maintaining a one-legged standing position with the dominant leg. The participants were instructed to keep their hands on their hips and the heel of the stance leg side in contact with the footplate while performing each reach. The maximum reach was measured by reading a tape measure at the end of the reach indicator, which reflected the point reached by the most distal part of the foot. If a participant failed to maintain the one-legged standing position, kicked the reach indicator, supported the body weight with the reach indicator, or failed to return to the reach foot at the center of the foot plate, then the attempt was discarded and the task was repeated. Three consecutive reach attempts were performed in the following order: forward, backward–inward, and backward–outward. The largest value of the reach distance in each direction was used for the analysis. The reach distances were normalized by the lower limb length (reach distance/limb length × 100) (15, 17). The lower limb length was measured (in cm) from the anterior superior iliac spine to the most distal part of the medial ankle using a cloth measuring tape. The composite reach score was calculated as the sum of the three reach distances divided by three times the limb length and multiplied by 100 (17, 18). Before the actual measurements were performed, each participant practiced one time under for each measurement condition. The measurements were performed in the following order: in the standard Y-Balance test; after a 5-minute rest period to account for fatigue; and in the Y-Balance test with the sensor game. The Y-Balance test was performed first while standing only on the dominant leg and then while standing only on the non-dominant leg during both tasks.

### Sensor game

The “Digireha” application (manufactured by Digireha Inc.), which utilizes an acceleration sensor (M5StickC Plus2; M5Stack) comprised the sensor game. “Digireha” is a gamification application that utilizes acceleration sensors, eye input sensors, voice sensors, and multiple infrared sensors; it has been introduced at pediatric facilities and facilities for the aged in Japan.

**Figure 2:**
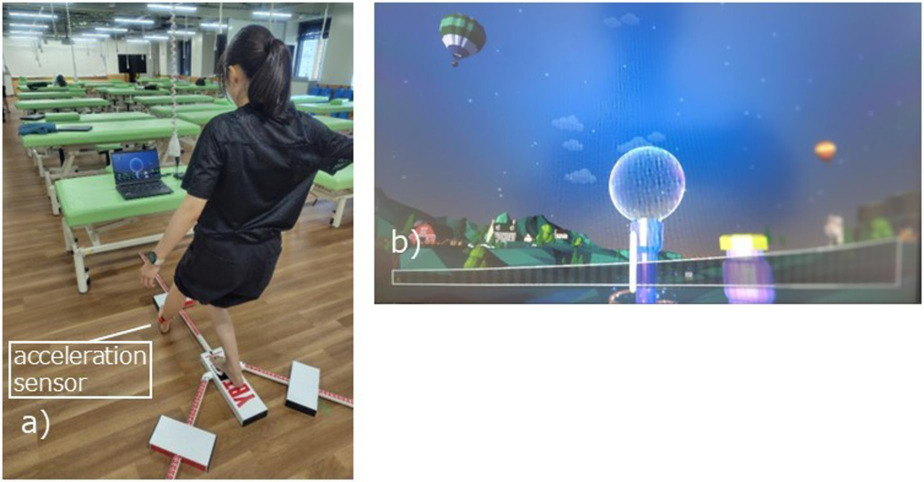
Scenes from the sensor game. **(a)** Performing the Y-balance test while playing “SoapBubble”. **(b)** “SoapBubble” screen.

In this study, the Y-Balance test was conducted by attaching an acceleration sensor to the foot that was to be reached during the Y-Balance test while playing “SoapBubble” (Figure 2). The accelerometer was secured with a belt 3 cm above the lateral malleolus of the fibula, with the sensor facing outward. “SoapBubble” is an application that has a set time during which a soap bubble forms; when the set time elapses, the bubble floats to the top of the screen, thus clearing the game. During game play, if the player moves faster than the speed set by the accelerometer, then the soap bubble on the screen breaks and the game restarts from the beginning. In this study, the set time for soap bubble formation was 60 s. Regarding the difficulty level of the task, the Y-Balance test was conducted with the accelerometer's sensor sensitivity set to 10 (0.768 g and 7.53 m/s^2^). The range of the sensitivity settings in the application was 1–20 (corresponding to 0.2 g and 1.96 m/s^2^–1.38 g and 13.5 m/s^2^); 20 was the most sensitive setting. The evaluator thoroughly practiced operating the equipment before the test was conducted. Because the acceleration sensor does not require calibration, the evaluator confirmed the connection between the computer and the accelerometer during each measurement before conducting the test. Under the aforementioned measurement conditions, participants first confirmed the speed at which the soap bubble burst and the speed at which it did not burst; then, they performed the Y-Balance test.

### Statistical analysis

In the main analyses of this study, to assume normality, the normality of all the variables was confirmed using the Shapiro–Wilk test, as well as visual assessment using histograms and Q-Q plots. An unpaired t-test was used to compare age, height, weight, and lower limb length of the participants, and Fisher's exact test was used to compare sex and the dominant leg. The results of the Y-Balance test were examined using a repeated measures two-way ANCOVA and a multiple comparison test using the Bonferroni method. In the ANCOVA, the presence of sensor games was analyzed as a within-subjects factor, and the difference between elementary school students and university students was analyzed as a between-subjects factor while controlling for age as a covariate. Statistical analyses were performed using IBM SPSS Statistics version 30, and the significance level was 5%.

## Results

The characteristics of the participants are listed in Table 1. The results of the repeated measures two-way ANCOVA of the presence or absence of the sensor game for each group are shown in Table 2. During the Y-Balance test of the dominant leg, tests of between-subjects effects revealed that age (as a covariate) was not statistically significant for any of the four items. Regarding the anterior direction, a main effect was observed between groups. No main effect of the presence or absence of the sensor game was observed, and no interaction was observed. Regarding the posteromedial, posterolateral, and composite scores, no main effect of the presence or absence of the sensor game was observed, and no main effect between groups was observed; additionally, no interaction was observed.

**Table 1:** Characteristics of the participants.

	All students (*n* = 79)	Elementary school students (*n* = 43)	University students (*n* = 36)	*p* value
Age, years, mean (SD)	14.4 (6.1)	9.0 (1.7)	20.8 (0.7)	<0.001*
Sex, male and female participants, *n*	36, 43	17, 26	19, 17	0.239
Dominant leg, right and left, *n*	77, 2	42, 1	35, 1	0.899
Height, cm, mean (SD)	148 (19.7)	133 (12.7)	165.9 (8.2)	<0.001*
Weight, kg, mean (SD)	43.5 (16.6)	31.6 (11)	57.8 (8.9)	<0.001*
Spina malleolar distance, cm, mean (SD)	70.1 (13.7)	59.2 (7.7)	83.1 (4.9)	<0.001*

* Elementary school students vs. university students.

* *p* < 0.05. SD, standard deviation.

**Table 2:** Analysis of variance results of for each parameter

		*F* value	Degrees of freedom	*p* value	*η*²ₚ
Y-Balance test of the dominant leg					
Anterior	Presence or absence of sensor game	0.716	1	0.400	0.009
	groups	13.492	1	<0.001*	0.151
	Age	0.195	1	0.660	0.003
	Presence or absence of sensor game × groups	0.012	1	0.911	<0.001
Posteromedial	Presence or absence of sensor game	0.143	1	0.707	0.002
	groups	0.059	1	0.809	0.001
	Age	1.667	1	0.201	0.021
	Presence or absence of sensor game × groups	0.106	1	0.745	0.001
Posterolateral	Presence or absence of sensor game	0.206	1	0.651	0.003
	groups	1.305	1	0.257	0.017
	Age	1.484	1	0.227	0.019
	Presence or absence of sensor game × groups	1.876	1	0.175	0.024
Composite score	Presence or absence of sensor game	0.075	1	0.785	0.001
	groups	3.940	1	0.051	0.049
	Age	1.386	1	0.243	0.018
	Presence or absence of sensor game × groups	0.283	1	0.597	0.004
Y-Balance test of the nondominant foot					
Anterior	Presence or absence of sensor game	0.001	1	0.976	<0.001
	groups	9.171	1	0.003*	0.108
	Age	0.089	1	0.767	0.001
	Presence or absence of sensor game × groups	0.776	1	0.381	0.010
Posteromedial	Presence or absence of sensor game	0.287	1	0.594	0.004
	groups	2.127	1	0.149	0.027
	Age	1.661	1	0.201	0.021
	Presence or absence of sensor game × groups	0.069	1	0.794	0.001
Posterolateral	Presence or absence of sensor game	0.050	1	0.824	0.001
	groups	0.117	1	0.733	0.002
	Age	2.145	1	0.147	0.027
	Presence or absence of sensor game × groups	0.035	1	0.852	<0.001
Composite score	Presence or absence of sensor game	0.107	1	0.744	0.001
	groups	3.402	1	0.069	0.043
	Age	1.016	1	0.317	0.013
	Presence or absence of sensor game × groups	0.228	1	0.635	0.003

* *p* < 0.05.

For the Y-Balance test of the nondominant leg, age (as a covariate) was not statistically significant for any of the four items. Regarding the anterior direction, a main effect between groups was observed. No main effect of the presence or absence of the sensor game was observed, and no interaction was observed. Regarding the posteromedial, posterolateral, and composite scores, no main effect of the presence or absence of the sensor game was observed, no between-group effect was observed, and no interaction was observed.

The results of each parameter after controlling for age as a covariate are presented in Table 3. Multiple comparison tests showed no differences in any of the items of the Y-Balance test with or without the sensory game for the dominant and non-dominant legs in the university student group. In the elementary school student group, the posterolateral score of the dominant leg decreased from 140.1% to 118.6% with the use of sensor games [mean difference, 21.5; mean difference confidence interval (CI), 0.780–42.312; adjusted *p* = 0.042].

**Table 3:** Comparison of parameters before and after efforts.

	Normal Y-Balance test		Y-Balance test with the sensor game	
	Elementary school student group	University student group	Elementary school student group	University student group
Y-Balance test of the dominant leg				
Anterior, %	137.2 (120.3–154.1)^a^	71.5 (51.5–91.6)	127.9 (107.2–148.6)^a^	64.5 (39.9–89.0)
Posteromedial, %	107.4 (88.1–126.7)	106.0 (83.1–128.8)	108.3 (89.6–126.9)	100.9 (78.8–122.9)
Posterolateral, %	140.1 (120.2–160.0)	104.2 (80.7–127.8)	118.6 (98.8–138.3)^b^	113.1 (89.8–136.5)
Composite score, %	128.2 (113.3–143.2)^a^	93.9 (76.2–111.6)	118.2 (101.1–135.4)	92.8 (72.5–113.2)
Y-Balance test of the nondominant leg				
Anterior, %	135.7 (118.0–153.4)^a^	73.1 (52.1–94.1)	119.1 (98.5–139.6)	75.4 (51.0–99.8)
Posteromedial, %	134.3 (112.2–156.5)	104.9 (78.7–131.1)	122.0 (101.6–142.4)	100.1 (76.0–124.3)
Posterolateral, %	113.8 (91.4–136.2)	104.9 (78.4–131.4)	108.5 (88.4–128.7)	103.8 (79.9–127.6)
Composite score, %	127.9 (110.8–145.0)	94.3 (74.1–114.6)	116.5 (98.5–134.6)	93.1 (71.8–114.4)

Data are presented as the estimated marginal means (95% confidence interval).

a Elementary school student group vs. university student group.

b Presence of the sensor game vs. absence of the sensor game.

The anterior scores of the dominant leg of the elementary school student group were higher than those of the university student group according to the normal Y-Balance test (mean difference, 65.7; mean difference CI, 29.6–101.8; adjusted *p* = 0.001) and the Y-Balance test with the sensor game (mean difference, 63.5; mean difference CI, 19.4–107.6; adjusted *p* = 0.005). The anterior scores of the non-dominant leg of the elementary school student group were higher than those of the university student group according to the normal Y-Balance test (mean difference, 62.6; mean difference CI, 24.8–100.4; adjusted *p* = 0.001). The composite scores of the dominant leg of the elementary school student group was higher than those of the university student group according to the normal Y-Balance test (mean difference, 34.3; mean difference CI, 2.5–66.1, adjusted *p* = 0.035). However, scores of the Y-Balance test with the sensor game did not differ between groups.

## Discussion

In the present study, after controlling for age as a covariate, interaction effects of the outcome measures were not observed, but main effects of several outcome measures were observed. The results of multiple comparison tests indicated that dynamic balance performance was influenced differently in the two groups depending on the use of the sensor game. Specifically, the elementary school student group had significantly reduced posterolateral score when the sensor game was used; however, the university student group did not exhibit significant differences in test scores.

The Y-Balance test is a dynamic balance assessment; therefore, fatigue is unlikely to occur after several repetitions, and learning effects may lead to improved scores after the initial attempt. In this study, the conventional Y-Balance test was first performed using the dominant foot. Next, the conventional Y-Balance test was performed using the non-dominant foot. Finally, a 5-minute rest period was allowed. Subsequently, the sensor game-based Y-Balance test was conducted using the dominant foot; thereafter, it was conducted using the non-dominant foot. If learning effects occur because of the fixed test order, then the results of the second Y-Balance test using the sensor game should improve. However, the results indicated no differences with or without the sensor, and the posterolateral scores of the elementary school students decreased. This finding suggests that using the sensor game may have increased the difficulty of the balance test by imposing movement speed constraints and supports our initial hypothesis that the increased task difficulty induced by the sensor-integrated game would temporarily reduce the performance of elementary school students but not that of university students. Additionally, this finding suggests that sensor games can be used to appropriately modulate task difficulty for younger populations.

Information regarding the amount and type of feedback provided during training is considered critical to adjusting task difficulty, particularly for children who tend to benefit from more feedback and longer practice times compared with those required by adults (19). In this study, visual feedback through gamification was used to promote slower movement execution to enhance motor control. Methods used to encourage a certain movement speed include voice calls by assistants and the use of a metronome for motor control through auditory information (20). In this study, gamification-based visual feedback was used to encourage slow movement. For elementary school students, visual information feedback effectively improves learning skills during physical education (21). Additionally, the ability of toys with built-in gyro-sensors to improve upper extremity fine motor skills and movements (22), the importance of visual information, and the use of sensors are attracting attention. Sensor games can maintain motivation during rehabilitation (13, 14) and promote repetitive training (15). Therefore, the use of sensory games that allow the provided amount of information to be adjusted may be effective for elementary school students.

Balance exercises using sensory games can be applied to improve the motor skills of healthy elementary school children as well as those of elementary school children with developmental coordination disorders. Providing visual, auditory, and other feedback to children with developmental coordination disorders using equipment can effectively improve motor skill learning and performance (23); therefore, balance practice using sensory games may improve the motor skills of healthy elementary school children.

This study had some limitations. Because of its cross-sectional design, the results were limited to differences in the immediate performance. Therefore, it was not possible to infer long-term effects or causal relationships from the intervention, and direct application of these results to clinical rehabilitation should be implemented with caution. Several previous studies have examined the Y-Balance test results of healthy elementary school students and university students (24, 25). The Y-Balance test scores observed during the present study were generally higher than those observed during previous studies. These findings may be related to racial differences and differences in exercise habits. Therefore, research of different sensor settings and validation among several racial groups should be conducted. Additionally, potential bias related to equipment support provided by Digireha Inc. may have occurred.

## Conclusion

This study investigated the differences in the dynamic balance function of healthy elementary school students and university students attributable to the use of the Y-Balance test with and without a sensor-integrated gamification application designed for pediatric rehabilitation. Our hypothesis that the dynamic balance function of healthy elementary school students and that of university students would decrease and would not significantly differ, respectively, when the sensor game was used to encourage slow movements was supported by these findings. These results suggest that a sensor-integrated gamification application can effectively increase the task difficulty for elementary school students by promoting slower and more controlled movements. Therefore, sensor-integrated gamification applications may be useful for adjusting exercise difficulty for this population.

## Data Availability

The original contributions presented in the study are included in the article/Supplementary Material, further inquiries can be directed to the corresponding author
